# Giant Cell Tumor of the Sacrum With Secondary Changes of an Aneurysmal Bone Cyst: A Case Report

**DOI:** 10.7759/cureus.101356

**Published:** 2026-01-12

**Authors:** Akanksha Kalwaghe, Suresh Phatak, Kajal Mitra, Prashant Onkar, Sagar Gund

**Affiliations:** 1 Department of Radiodiagnosis, N. K. P. Salve Institute of Medical Sciences and Research Center, Nagpur, IND

**Keywords:** aneurysmal bone cyst, ct scan, giant cell tumor, mri spine, neurological symptoms

## Abstract

Giant cell tumor (GCT) of bone is a typically benign but locally aggressive neoplasm that occurs between the ages of 20 and 40 years, and most commonly arises in the epiphysis of long bones, with sacral involvement being rare and diagnostically challenging due to its insidious onset and nonspecific symptoms. We report the case of a 21-year-old woman who presented with progressive low back pain radiating to both lower limbs, associated with perianal numbness and bilateral heel pain aggravated by physical activity. Imaging revealed a well-defined, expansile osteolytic lesion involving the S1-S2 vertebrae with features suggestive of a GCT. Magnetic resonance imaging (MRI) demonstrated an avidly enhancing mass extending into the spinal canal, causing compression of the thecal sac and adjacent nerve roots, more pronounced on the right side, along with fluid-fluid levels consistent with secondary aneurysmal bone cyst-like changes. Histopathological examination confirmed the diagnosis of GCT with secondary aneurysmal bone cyst transformation. Sacral GCTs are rare, locally aggressive lesions that often present with neurological symptoms, and imaging typically shows an expansile “soap-bubble” osteolytic lesion with possible soft-tissue extension and secondary cystic changes. Surgical excision remains the mainstay of treatment, with preoperative transarterial embolization playing an important role in reducing tumor vascularity and intraoperative blood loss. This case highlights the importance of including GCT in the differential diagnosis of sacral lesions in young adults and underscores the role of timely imaging and biopsy in establishing an accurate diagnosis and guiding appropriate management.

## Introduction

Giant cell tumor (GCT) of bone is a benign yet locally aggressive tumor that comprises about 5% of all primary bone tumors, most commonly affecting skeletally mature young adults [1]. On histology, it demonstrates multinucleated osteoclast-like giant cells mixed with mononuclear stromal cells [2]. These tumors typically arise in the epiphysis and metaphysis of long bones, most frequently involving the distal radius, distal femur, and proximal tibia [3]. Spinal involvement is rare (approximately 1.7-8.2% of cases), with the sacrum being the most frequently affected site within the axial skeleton [4,5].

GCTs of the sacrum are challenging to diagnose due to their insidious onset and non-specific symptoms, and also mimic other spinal pathologies [6]. Patients commonly present with low back pain, radiculopathy, and neurological deficits secondary to nerve root or thecal sac compression. The anatomical complexity and deep location of the sacrum contribute to delays in clinical evaluation and imaging.

Secondary aneurysmal bone cysts (ABCs) are blood-filled and show expansile cystic changes that can develop within pre-existing bone tumors such as GCTs [7,8]. Unlike primary ABCs, which are associated with gene rearrangements (USP6), secondary ABCs occur due to degenerative transformations within other bone pathologies, including GCT, osteoblastoma, chondroblastoma, or fibrous dysplasia [8]. In 14-20% of cases, the coexistence of secondary ABC components in GCTs is believed to contribute to faster growth, cortical destruction, and a higher risk of structural compromise or neurological symptoms due to pressure effects [9]. On MRI, these lesions typically show a multiloculated appearance, showing fluid-fluid levels that represent hemorrhages at different stages of evolution [10]. Secondary ABC changes in GCT indicate increased vascularity and aggressive expansile behavior, influencing imaging interpretation, bleeding risk, and the need for preoperative embolization or adjunctive therapy [6,8,9].

In the spine, GCTs with secondary ABCs generally originate from the vertebral body, in contrast to primary ABCs that usually arise from the posterior elements and are more common in patients below 20 years of age [10]. On MRI or FDG-PET/CT, these lesions display both solid, enhancing, and cystic hemorrhagic areas, with the solid regions showing greater metabolic activity [11]. Identification of this combination is crucial, as it affects both diagnosis and therapeutic planning, further potentially guiding the use of embolization or denosumab therapy along with surgical management. Sacral GCTs show significant surgical challenges due to complex anatomy and proximity to neural, vascular, and visceral structures, limiting complete resection and increasing neurological morbidity and recurrence risk [4,5,12].

This case is interesting as it is rare and clinically complex and focuses on the importance of clinical, radiologic, and histopathologic assessments in the management of spinal tumors. There is limited literature on sacral GCTs with secondary ABC and highlights the need for high clinical suspicion in young adults presenting with atypical spinal symptoms.

## Case presentation

A 21-year-old woman presented with insidious, progressively worsening dull low back pain radiating to both lower limbs for four months, associated with numbness. There was no history of trauma, infection, or chronic medication use. Four days prior to presentation, she developed bilateral heel pain aggravated by standing and walking, along with recent-onset perianal numbness, raising concern for neurological involvement.

On examination, localized tenderness was noted over the sacral region (S1-S2). No swelling or palpable mass was present. Neurological examination showed normal motor strength (MRC 5/5) and preserved reflexes, with hypoesthesia limited to the perianal region. There was no bladder or bowel dysfunction. Systemic examination and lymph node assessment were unremarkable.

**Figure 1:**
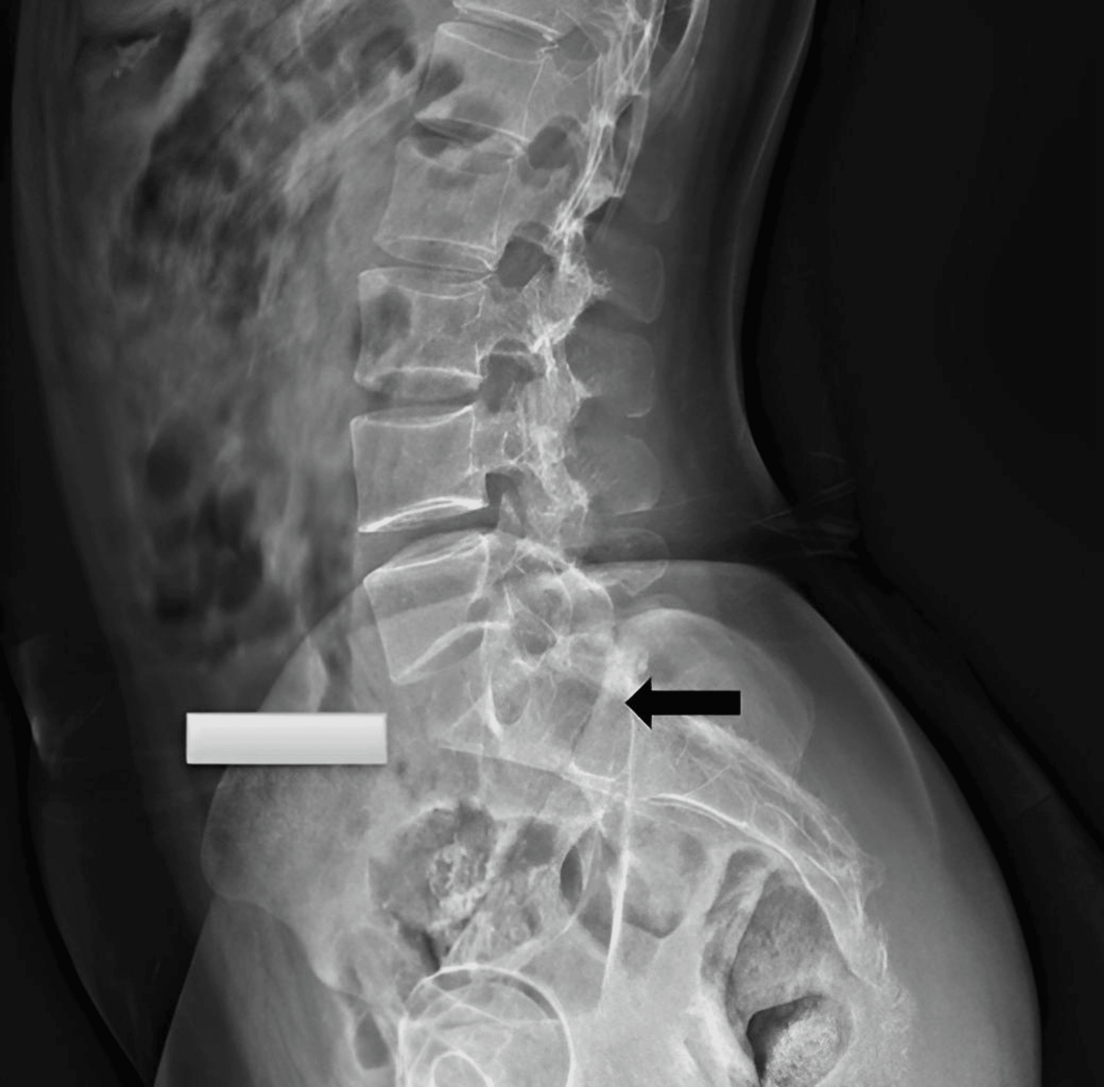
X-ray lumbar spine lateral view: An expansile, lytic lesion involving the S1 and S2 vertebral bodies (black arrow) demonstrated characteristic "soap-bubble" radiolucency with cortical thinning.

Radiographic evaluation included a plain X-ray of the lumbosacral spine, which revealed a lytic lesion involving the S1 and S2 vertebral bodies. The lesion demonstrated characteristic "soap-bubble" radiolucency with cortical thinning and an ill-defined margin (Figure 1). 

**Figure 2:**
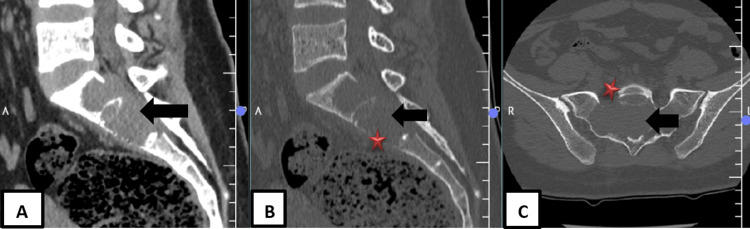
(A) Sagittal soft-tissue CT, (B) sagittal bone-window CT, and (C) axial bone-window CT at sacral level shows a well-defined expansile osteolytic lesion centered in S1–S2 (black arrow) with marked endosteal scalloping, cortical thinning, and mild anterior cortical breach at S2 (red star). The lesion has a narrow zone of transition without periosteal reaction and extends posteriorly into the spinal canal, causing mass effect on the thecal sac.

Computed tomography (CT) of the lumbosacral spine delineated a well-defined, expansile, osteolytic lesion centered in the S1 and S2 vertebrae. The lesion caused significant endosteal scalloping, cortical thinning, and mild cortical breach anteriorly at S2. It had a narrow zone of transition without evidence of periosteal reaction. A heterogeneous soft tissue component was seen extending posteriorly into the spinal canal, exerting mass effect on the thecal sac (Figure 2A-2C).

**Figure 3:**
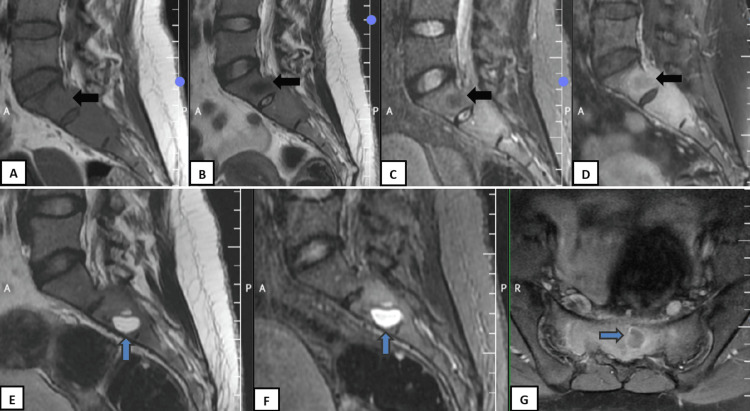
A panel of MRI images showing sagittal (A) T1WI, (B) T2WI, (C) STIR, (D) T1 post contrast, (E) T2WI, (F) STIR, (G) Axial T1 post contrast. A small ill-defined area (black arrow) is noted within S1 appearing T1 isointense and T2/STIR hypointense, demonstrating mild post-contrast enhancement, suggestive of solid tumor components. The cystic component (blue arrow) is hyperintense on T2-weighted imaging (T2WI) and short-tau inversion recovery (STIR) sequences and peripherally enhancing on the post contrast study.

Magnetic resonance imaging (MRI) with contrast of the lumbosacral spine revealed a well-defined expansile lesion measuring 2.3 x 6.9 x 5.1 cm (AP x TR x CC) involving S1, S2, and the posterosuperior corner of S3 with mild extension into both sacral alae. A small solid component in S1 was T1 isointense and T2/STIR hypointense and showed mild enhancement. Multiple thick-walled, peripherally enhancing cystic lesions within S1-S2 were T1 hypointense and T2/STIR hyperintense. The lesion extended into the spinal canal, causing moderate thecal sac and cauda equina nerve root compression (right > left), without presacral or paraspinal muscle involvement or cortical breach (Figure 3A-3G).

CT-guided core needle biopsy showed fibroblastic stroma with numerous osteoclast-like multinucleated giant cells (10-15 nuclei) and low mitotic activity. Areas of hemorrhage and blood-filled cystic spaces with fibrous septa were present, consistent with secondary ABC changes. No granulomatous inflammation was identified.

Based on the clinical presentation, imaging features, and histopathological findings, a final diagnosis of GCT of the sacrum with secondary ABC was established. The patient underwent an interventional treatment. After treatment, there was improvement in the patient’s clinical condition in the form of pain relief and sensory recovery.

Treatment

Post biopsy, denosumab** **was given as a loading dose of 120 mg (subcutaneous injection), followed by a continuous dose on days 1, 8, 15, and 29 and then every four weeks.

**Figure 4:**
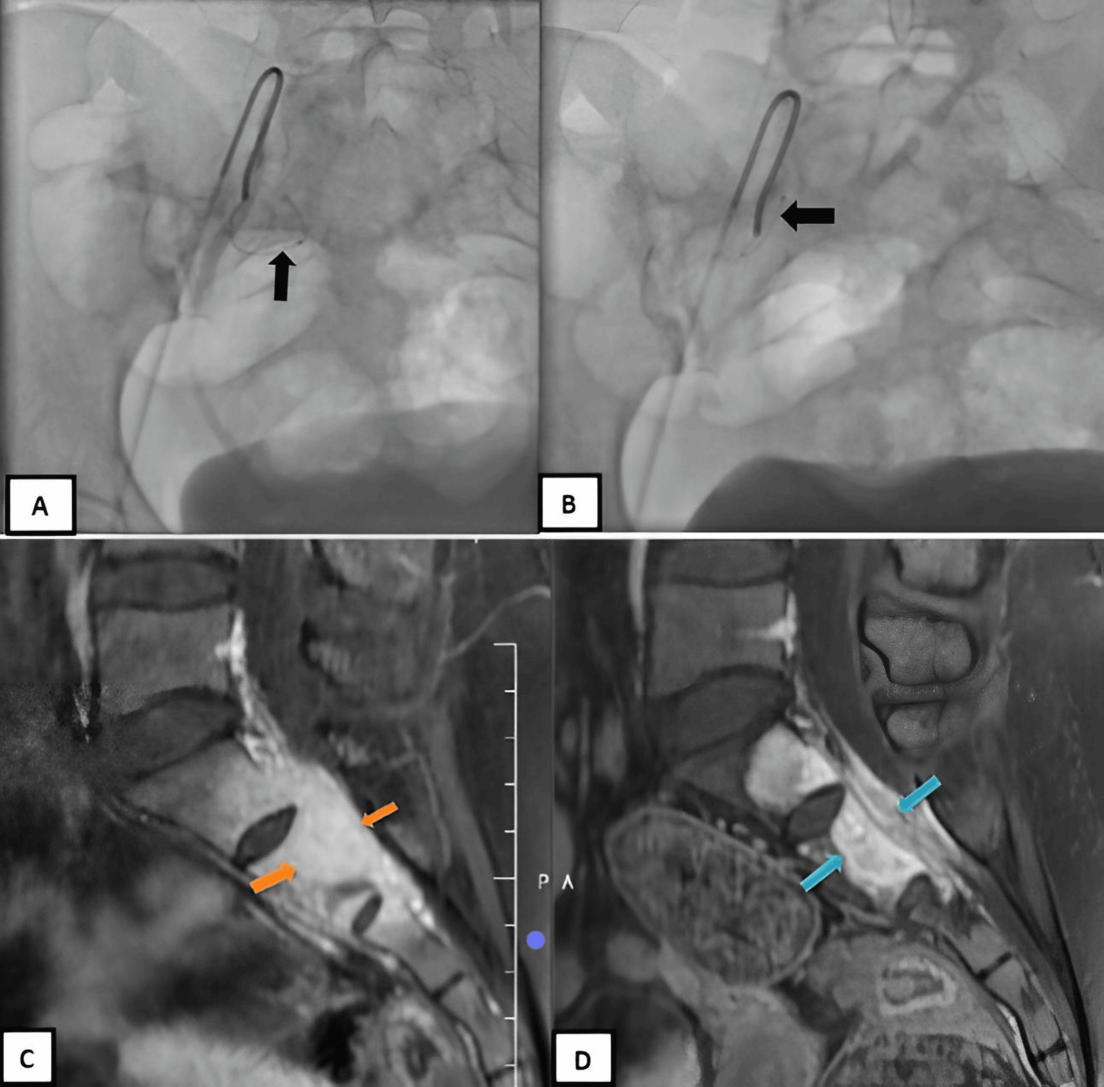
DSA AP view (A, B): Selective angioembolization of branches of the right internal iliac artery supplying the tumor. The branches were individually cannulated (black arrow) and embolized using 300-500 u PVA particles. (C, D) MRI Sag T1 FS + C before and after denosumab therapy and angioembolization revealed reduction in the AP diameter (orange and blue arrows) of the lesion with reduction of the cauda equina compression but the same contrast enhancement as above, suggesting partial resolution.

Two months after the denosumab therapy,embolization was performed via right common femoral artery (6F) access. Bilateral internal iliac angiography demonstrated two tumor-feeding branches from the right internal iliac artery, which were selectively cannulated (Figure 4A-4B) and embolized with 300-500 µm polyvinyl alcohol particles. Post-embolization angiography showed successful occlusion of the feeding vessels with preservation of the remaining internal iliac branches (Figure 4A-4B). No therapy-related complications were noted in the patient.

Follow-up MRI after six months revealed a reduction in the size of the lesion to 1.6 x 6.3 x 4.4 cm (AP x TR x CC) and mild reduction of the cauda equina compression with a slight decrease in the enhancement of the lesion and low signal intensity at the periphery, suggesting marginal sclerosis (Figure 4C-4D).

## Discussion

GCT of bone is a primary neoplasm with distinctive histological features, marked by the multinucleated giant cells scattered within a mononuclear stromal background [2]. While it predominantly affects the appendicular skeleton in skeletally mature individuals aged 20-40 years, axial involvement, especially of the sacrum, is infrequent [4,5].

Sacral GCTs are diagnostically challenging due to their deep location and vague symptomatology, often mimicking more common conditions like mechanical back pain, disc herniation, or spinal infections [6,10]. Neurological symptoms such as radiculopathy, paresthesia, and sphincter dysfunction occur due to direct compression or invasion of sacral nerve roots [11]. This patient presented with perianal hypoesthesia and heel pain, correlating with S1-S2 nerve root involvement.

Radiographically,sacral GCTs present as expansile osteolytic lesions with a multiloculated “soap-bubble” appearance due to residual trabeculae [3,12]. Cortical thinning and expansion are common, with occasional focal breaches. OnCT, they appear as non-mineralized soft-tissue density masses with variable attenuation from hemorrhage or necrosis and may show a thin eccentric sclerotic rim opposite areas of cortical erosion.

MRI delineates the extent of sacral GCTs into adjacent soft tissues, spinal canal, and neurovascular structures. The solid components are typically hypo- to isointense on T2-weighted images due to collagen and hemosiderin, with vivid post-contrast enhancement reflecting high vascularity, while cystic or hemorrhagic areas appear hyperintense. Multiple cystic spaces with fluid-fluid levels and non-enhancing septa suggest a secondary ABC component. On ¹⁸F-FDG PET/CT, solid tumor areas show higher tracer uptake than cystic regions [9].

Bone scintigraphy in GCT with secondary ABC-like changes typically shows heterogeneous uptake with increased peripheral activity and reduced central uptake, reflecting active bone formation at the margins and cystic or hemorrhagic components centrally [9].

On ¹⁸F-FDG PET/CT, there is heterogeneous FDG uptake, with higher uptake in solid tumor components and low or absent uptake in cystic/aneurysmal areas, helping distinguish viable tumor from secondary changes [9].

Treatment strategies for sacral GCTs depend on lesion size, location, and neurological involvement. Treatment options are intralesional curettage, en bloc resection, arterial embolization, and adjuvant therapies like cryotherapy, phenol, and denosumab. Denosumab may cause hypocalcemia, osteonecrosis of the jaw, and treatment-related sclerosis that can obscure residual tumor and influence recurrence assessment [13,14]. When in proximity to neurovascular structures, wide resection in the sacrum is often not feasible; in such cases and treatment aims to achieve local control while preserving neurological function.

Preoperative trans-arterial embolization (TAE) reduces intraoperative hemorrhage in highly vascular bone tumors by selectively catheterizing tumor feeders and deploying embolic agents such as polyvinyl alcohol particles, gelatin sponge, or coils. Post-embolization angiography shows marked reduction or disappearance of tumor blush, indicating effective devascularization, thereby facilitating safer surgery with reduced blood loss, improved visualization, and lower transfusion requirements. Trans-arterial angioembolization can be complicated by non-target embolization, post-embolization pain, and ischemic or neurological deficits, particularly in spinal and sacral lesions [15].

Given the known risk of local recurrence in GCT of bone, particularly in axial and sacral locations, long-term outcome and recurrence assessment cannot be commented upon at present as the patient is currently undergoing treatment, which represents a limitation of this report.

Differentials

Infection (Osteomyelitis) 

It is a lytic destructive lesion with marrow edema and adjacent soft-tissue or paravertebral collections, mimicking sacral GCT [4,5,10].

Chordoma (Sacral Location)

It is a midline sacral mass with bone destruction and soft-tissue component, typically showing different imaging characteristics and patient demographics [4,10].

Metastatic Disease

It may mimic sacral GCT on imaging but often presents with multiple lesions and a known primary malignancy [4,10].

## Conclusions

This case focuses our attention on the uncommon and often challenging nature of sacral GCTs, especially when they undergo change like a secondary ABC. The patient’s neurological symptoms, along with the imaging and histologic findings, helped establish the diagnosis with precision. It is important for clinicians to consider this possibility in young adults who present with persistent lower back pain and neurological complaints, even when systemic signs or striking radiologic changes are absent. Using a combination of imaging techniques and confirming the findings through tissue diagnosis remain essential for planning management. A limitation of this report is the short follow-up duration, as the patient is currently undergoing treatment, necessitating a long-term surveillance. By presenting this case, we hope to add to the limited number of reports on sacral GCTs and to stress how early recognition and collaboration across specialties can influence the outcome.
